# Surgery of the Liver and Gall Bladder

**Published:** 1895-05-25

**Authors:** 


### SURGERY OF THE LIVER AND GALL BLADDER.

**Partial Extirpation of the Liver.**—Israel[1] reports a case in which the patient, aged fifteen years, died of numerous metastases three months after a primary sarcoma of the liver had been removed. Before operation it could not be determined whether the tumour was of hepatic or renal origin, but on opening the abdomen it was found to spring from the right lobe of the liver, and there were no adhesions, except below, to the omentum. The growth was removed from right to left by the cautery knife. The hæmorrhage was arrested by pressure with gauze. The amputation wound in the liver was about fifteen centimetres, and the tumour removed weighed 1,225 grains, and was proved to be an angio-sarcoma. The author looks upon the results obtained by operation in fourteen cases of hepatic tumours as encouraging. Six of these were syphilitic, and of these two died. He considers that a single operation, either with the extra peritoneal treatment of the liver wound or with the use of the tampon, is the best. The former is more desirable, but it is not always practicable. Elsberg's method of fastening the tampon with stitches provides the greatest security against hæmorrhage. The simple packing, as practised in the above case, is not so certain, but secondary bleeding can then readily be recognised. Where constriction by tubing is possible the knife is preferable to the cautery as the large vessels can be surrounded or tied. Tricomi[2] has removed a tumour the size of a man's fist from the left lobe of the liver. Attempts to remove the growth by ligature were unsuccessful, and the growth was ultimately cut away and bleeding stopped by the thermo-cautery and iron solution. In this relation, Ceccherelli and Bianchi's method of arresting hæmorrhage from the liver is of interest. This consists in the application of a quilled suture. The portion of the liver which it is proposed to excise is circumscribed by two strips of whalebone, each perforated from end to end by several holes. A long needle armed with a double thread is passed through the hole at one end of one strip of whalebone, then through the liver structure, and finally through the hole at the corresponding end of the second strip. Other ligatures are passed through all the other holes in both strips, and through the intervening portion of liver. One of the ends of the first ligature is tied to the cut end on the opposite side, and then all the ends are closely tied together along the whole length of one strip of whalebone, and afterwards along the second strip. In this way the interposed liver tissue is closely constricted and gradually crushed. All the ligatures having been secured, the liver may be freely incised without dread of hæmorrhage.

**Wounds of the Liver.**—Zeidler[3] observes that the diagnosis of ruptures of the liver is not easy, but laparotomy is indicated here as in the wounds of other viscera. Such symptoms as local and radiating pains, icterus, enlarged and tender liver arise late, and yet the danger lies within the first twenty-four hours in the majority of cases. The author has successfully treated three cases by abdominal section. It is striking that small wounds in the liver can produce so much hæmorrhage. The tendency to spontaneous arrest of the hæmorrhage is slight. Suture of the liver, Paquelin's cautery, and the tampon are the methods available. The blood-pressure in the liver vessels is low, hence the arrest of hæmorrhage can surely be obtained by the tampon. The wound in the liver where the tampon is used also remains better under observation.

**Hydatid Disease.**—Ross[4] reports thirteen cases of hydatid disease of the liver, which he divides into three sets—(1) Those springing from the posterior, superior, or antero-superior aspect, eight cases; (2) from the inferior aspect, four cases; (3) intra-hepatic, one case. The majority of them were operated upon by Volkmann's method, which the author recommends to the general practitioner, as skilled assistance is not required. Whenever a cyst was located on the convexity of the liver nerve pressure symptoms were noticed, and sometimes they were the only symptoms complained of by the patient. He advises that the adventitious cyst should be removed whenever possible, as this hastens recovery, and prevents complications. Cysts on the convexity of the liver are best operated upon through the diaphragm.

**The Anatomy of the Right Hypochondrium in Relation to Operations for Gall Stones** is the subject of a paper by Morison,[5] who concludes that a pouch exists below the right lobe of the liver and gall bladder, separated from the general peritoneal cavity by natural barriers. This pouch can be efficiently drained through an opening in the parietes near the lower end of the kidney. A transverse is better than a vertical incision in operating for gall stones; less likely to be followed by ventral hernia and giving free access. Biliary fistula results from operations for gall stones in a considerable percentage of cases in which the gall bladder has been attached by sutures to the parietes. The method of attachment has little to do with this result, and it may follow when the ducts are patent. The gall bladder and ducts may safely be allowed to empty into the pouch described, if it is properly drained. The gall bladder should never, except when suppurating, be stitched to the abdominal wall. If the pouch is properly drained (*a*) when the gall bladder is distended the opening in it should be closed by sutures and the viscus returned into the abdominal cavity, and the drain left until the certainty of its successful closing is complete. (*b*) When the gall bladder is shrunken, and there is difficulty in closing the opening made in it, it may be returned unclosed. (*c*) When a stone is impacted in the cystic duct, and evades all ordinary efforts to remove it, the gall bladder should be excised and the duct ligatured after removing the stone in it. (*d*) When a stone is impacted in the common duct, the duct is incised, and after the stone or stones are removed, the opening may be left unclosed if there is any difficulty in applying a satisfactory suture.

[1] Brit. Med. Jour., Sept. 22, 1894, and Deut. Med. Woch, Aug. 23, 1894. [2] Therapeut. Gaz., Aug. 15, 1894, and Rev. de Chir., 1894. [3] Brit. Med. Jour., Oct. 13, 1894, and Deut. Med. Woch, Sept. 13, 1894. [4] Brit. Med. Jour., Sept. 22, 1894, and Austral. Med. Gaz., July 15, 1894. [5] Brit. Med. Jour., Nov. 3, 1894, p. 968.

